# Reducing Unwarranted Variation in Emergency Department Laboratory Test Ordering With a Feedback-Based Decision Support Tool: Qualitative Formative Evaluation

**DOI:** 10.2196/92744

**Published:** 2026-11-13

**Authors:** Julie Li, Craig Scowen, Conrad Loten, Guilherme Franco, Alex Eigenstetter, Judith Thomas, Melissa Baysari, Andrew Georgiou, Mirela Prgomet

**Affiliations:** 1 Centre for Health Systems and Safety Research, Australian Institute of Health Innovation, Macquarie University, 75 Talavera Road, Macquarie University, Sydney, New South Wales, 2109, Australia, 61 +61 (2) 9850 24; 2 New South Wales Health Pathology, Sydney, NSW, Australia; 3 Emergency Department, Hunter New England Local Health District, Newcastle, NSW, Australia; 4 Susan Wakil School of Nursing and Midwifery, The University of Sydney, Sydney, New South Wales, Australia

**Keywords:** emergency service, hospital, clinical laboratory techniques, decision support systems, clinical, qualitative research, quality improvement

## Abstract

**Background:**

Substantial unwarranted variations exist in laboratory test ordering practices in the emergency department (ED), despite a plethora of digital health interventions to facilitate laboratory test ordering consistent with best-practice recommendations. Drivers of such variation are many and varied and deeply ingrained within organizational culture and work practices. An appreciation of the impact of contextual factors on digital health implementation is essential for achieving intended outcomes.

**Objective:**

This study aimed to design, develop, and formatively evaluate a digital health tool to reduce unwarranted variation in pathology test ordering, the Emergency Department Pathology Ordering Support Tool (ED-POST), through co-design and collaboration with key stakeholders.

**Methods:**

A multimethod qualitative co-design approach with formative evaluation was undertaken across 3 phases in a large Australian ED between April and December 2023. Phase 1 involved semistructured interviews with ED clinicians and observations to examine existing workflows, decision-making processes, and user requirements, informing prototype development. Phase 2 included demonstrations of the ED-POST prototype to ED clinicians, followed by semistructured interviews to explore perceived usability, utility, and impact. Phase 3 comprised a stakeholder forum with clinicians, researchers, and developers to assess scalability and refine design. Participants were selected purposively, and data were analyzed inductively to generate themes.

**Results:**

The perceived use and value of ED-POST varied depending on user role. Triage nurses who ordered preliminary tests at the beginning of the patient encounter were more likely to use ED-POST for point-of-order decision-making. Junior physicians saw value in ED-POST as a form of backup to reaffirm personal impressions. Senior physicians responsible for determining a differential diagnosis at a later stage of the patient journey saw less utility in ED-POST for supporting ordering decisions but saw potential in its big-data synthesis capabilities to support reporting and quality improvement.

**Conclusions:**

Understanding roles and workflows in the early developmental stages of a digital health tool and user involvement at every stage are necessary to optimize adoption, scalability, and outcomes. Future digital tool design and implementation initiatives should focus on feedback-driven mechanisms with real-time data integration to facilitate more informed ordering behaviors.

## Introduction

The emergency department (ED) is a unique clinical environment that manages an unpredictable case mix of acute, traumatic, or life-threatening conditions. Laboratory investigations play an important role in the evaluation of undifferentiated presentations in this time-sensitive setting. Despite a plethora of digital health-driven interventions to facilitate laboratory test ordering consistent with best-practice recommendations in the ED, substantial unwarranted variations remain across hospitals and clinicians [1-3]. Addressing unwarranted variation in health service provision is an essential element in quality improvement, as unwarranted variation in laboratory test use may be indicative of undertesting, which can lead to missed diagnoses [4], or overtesting, which can result in an excess of added investigations resulting in potential risk to patients [5].

Unwarranted variation in ED laboratory test ordering refers to inconsistent laboratory requests that are not justified by patient medical need, severity of illness, or preference [6]. Examples include routine ordering of broad test panels (eg, full blood count, electrolytes, and liver function tests) for low-acuity presentations [7], variation in testing without adherence to validated clinical decision rules [8], and frequent repeat testing within short intervals without clear clinical justification [9]. Evidence suggests that up to one-third of laboratory tests ordered for patients in ED presenting with undifferentiated chest pain may not align with guideline recommendations [10]. Similar patterns of variation have been documented internationally across a range of health care contexts, including primary care, inpatient medicine, and intensive care settings, where studies have demonstrated wide interprovider and interinstitutional differences in ordering rates for common tests. Such variation has also been reported in health systems with sophisticated digital health infrastructure, suggesting that the presence of technology alone is insufficient to standardize practice [11,12].

The effectiveness of digital health interventions is highly dependent on contextual factors, including usability, integration into clinical workflows, and stakeholder engagement [13]. Systematic reviews and meta-analyses of clinical decision support systems show modest improvements in care outcomes, suggesting that intervention effects are context-dependent and not uniformly sustained, particularly when not tailored to local contexts [11,12].

The underlying drivers for unwarranted variation in laboratory test ordering might be many and varied within and across care settings and deeply ingrained within organizational culture and work practices. A broad appreciation of the impact of contextual factors on digital health implementation is essential to ensure that intended tools are used and outcomes are achieved following implementation [14].

A number of state and national initiatives in Australia aimed to decrease unwarranted variation in test ordering practices but relied on manual processes that were difficult to sustain [15,16]. This study aimed to design, develop, and formatively evaluate a digital health tool for reducing unwarranted variation in pathology test ordering, the Emergency Department Pathology Ordering Support Tool (ED-POST), through co-design and collaboration with representatives from hospital, laboratory, and health services research sectors.

## Methods

### Design and Setting

This study used a multimethod qualitative co-design approach [17] as a way of understanding existing pathology test ordering and decision-making workflows to ensure that the final digital tool was suitable for the clinical context and that user needs were considered at every phase of development. Formative evaluation was also undertaken during the development of the digital tool for the purpose of system improvement [18]. The co-design and evaluation of the decision support tool were conducted across 3 phases.

Phase 1 comprised in-depth interviews and an observation session to understand current laboratory test ordering workflows and issues, decision-making requirements, and expectations for an electronic decision-support tool. Findings from this phase were presented to developers of the tool [19] (laboratory management, a data scientist, and a health services researcher) who created the ED-POST prototype.

Phase 2 comprised demonstrations of the prototype to individual clinicians and the collection of clinician feedback via semistructured interviews. Findings from phase 2 were triangulated at a stakeholder forum during phase 3, which included developers of the tool and representatives from 2 additional EDs in the same public hospital network, to explore the applicability of the tool across hospitals and areas for further development. A summary of each phase is presented in Figure 1.

The study was conducted in the ED of a major regional teaching hospital and Level 1 Trauma Center in New South Wales, Australia. Hospital demographics are presented in Table 1. This hospital was selected based on its designation as the intended pilot site for the intervention.

**Figure 1.**
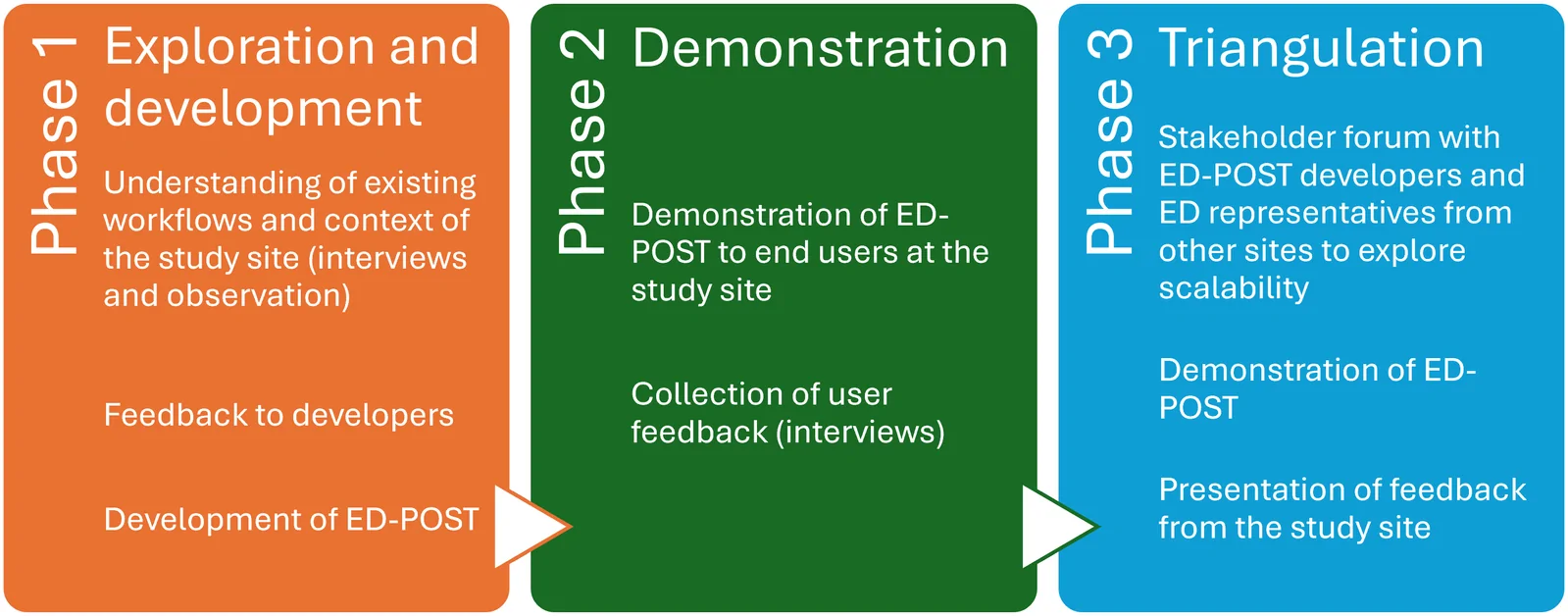
Co-design and evaluation phases. ED: emergency department; ED-POST: Emergency Department Pathology Ordering Support Tool.

**Table 1.** Study site demographics.

Site characteristic	Total, n
Number of beds	796 [20]
ED^a^ beds	42 [21]
ED attendances per year	83,671 [22]
Admitted patient episodes of care per year	26,120 [22]

a ED: emergency department.

### Ethical Considerations

Ethics approval for the project was granted by the human research ethics committee of the relevant local health district (2022/ETH01809). Written informed consent was obtained from all participants via a signed formal consent form as part of a study information pack. All collected data were deidentified and stored electronically in a secure cloud-based location accessible only to project study team members. Participants were not compensated for their participation in the study.

### Data Collection

In phase 1, semistructured interviews were conducted with key informants (2 senior physicians, a senior nurse, and a mid-level physician) to understand the pathology test ordering and decision-making workflow and requirements. Interviews took place in April 2023 (range: 16 minutes 45 seconds to 1 hour 2 minutes 33 seconds), with participants purposively selected based on their involvement in and familiarity with the laboratory test ordering process. This criterion was relevant to this study, as the included participants were the intended users of the prototype. A senior ED physician assisted with identifying and inviting relevant candidates to participate. Interviews were supplemented with a 30-minute observation session in the ED triage area, where the majority of test ordering took place. Observational field notes, nonidentifiable photographs of relevant artifacts, and documentation (eg, guideline documents and blank laboratory test order forms) were also collected to provide further context. Findings from this phase informed the development of the ED-POST prototype.

In phase 2, individual demonstrations of the tool were delivered onsite in October 2023 to a larger sample of frontline clinicians (n=11). Criterion-based purposive sampling [23] was reused in this phase, in which a senior ED physician invited relevant participants based on their knowledge of and engagement with the laboratory test ordering process as a formal component of their clinical role. Immediately postdemonstration, participants were asked to describe the usability, utility, and potential impact of the tool on decision-making and workflow via semistructured interviews (range: 4 minutes 21 seconds to 13 minutes 23 seconds) and to offer any suggestions for improvement. Interview questions included perceived usefulness for ordering tasks, impact on workflow (speed/efficiency and sequence/coordination/number of process steps), influence on decision-making, and recommendations.

In phase 3, outcomes of the demonstration and of the ED-POST tool were presented at a 1-hour forum discussion to stakeholders external to the study site in December 2023. Developers of the ED-POST prototype were invited to attend, and the same senior ED physician who recruited for phases 1 and 2 recruited ED directors from 2 hospitals in the same geographic area. During the resulting stakeholder forum, findings from phase 2 were presented to developers and triangulated with those external clinicians, and the applicability and scalability of ED-POST across sites were explored. Interview and discussion guides for all phases of the study are presented in Multimedia Appendix 1.

### Data Analysis

Interviews and observations from phases 1 and 2 were audio-recorded and transcribed verbatim for inductive analysis using a general inductive approach to allow findings to emerge from the data and to avoid the constraints of preexisting theories or hypotheses [24]. Minutes were recorded during the stakeholder forum in phase 3 and analyzed in the same manner. Analysis was conducted by an experienced qualitative researcher (JL) who also performed all data collection but had no previous engagement with the study site. Transcript data from all 3 phases were analyzed to develop themes using an inductive, iterative process of initial open coding, constant coding review, and development of categories from open codes [24]. Final themes were presented to the broader research team and reviewed by a key informant from the site for member checking to establish trustworthiness and credibility of findings.

### Business Process Modeling and Notation

In this study, the Object Management Group’s Business Process Model and Notation (BPMN) [25] was used for mapping work processes in phase 1. BPMN is a standard graphical representation of work processes in a process diagram and plays a crucial role in bridging communication gaps between stakeholders [26]. BPMN’s standardized and intuitive graphical nature enables shared understanding between technical and other stakeholders and supports activities such as process design, implementation, monitoring, and management [26]. BPMN has been used to document the clinical context for digital health at the level of tasks, resources, and workflows and is valuable to the efficacious implementation of digital health [27]. In this study, BPMN was used as a means of presenting contextual workflows to ED-POST developers. Information extracted from interviews was used to identify the tasks, roles, and information requirements of the intended users of the prototype and presented to ED-POST developers alongside the prototype development document (Multimedia Appendix 2).

## Results

### Overview

Demographics of participants across all phases of the study are presented in Table 2. In phase 1, clinicians described existing laboratory ordering work practices, which were mapped and presented to the team that developed the ED-POST prototype. Clinicians’ perceptions on issues, ordering decision-making needs, and expectations for a digital ordering tool were compiled in a prototype development document (Multimedia Appendix 2). A subset of preliminary findings from this phase has been previously published [19]. Across phases 2 and 3, participants responded to demonstrations of the ED-POST tool, raising insights into the following themes: (1) the impact of ordering role on the perceived use and value of the ED-POST tool, (2) occupational hierarchy, role-based priorities, and their broader implications for the ED-POST tool, (3) drivers of test ordering behavior, and (4) the real cost of laboratory testing.

**Table 2.** Participant demographics.

Study phase and role	Total, n
Interviews	
Advanced practice nurse	2
Senior physician	4
Registered nurse	2
Junior/mid-level physician	7
Forum	
Researcher	2
Data scientist/IT	1
ED^a^ director	2

a ED: emergency department.

### Clinician Test Ordering Work Processes (Phase 1: Exploration)

In the absence of an electronic medical record (EMR) at the study site, participants reported that the requesting of laboratory tests remained paper-based. A site-specific characteristic of the ordering workflow compared with other hospitals was the expanded role of registered nurses who held more autonomy and ordering rights (within the scope of a locally developed guideline) and performed the majority of laboratory test ordering for the department at patient triage. Once a paper pathology request form was completed, it was sent with a blood sample to the laboratory via pneumatic chutes situated throughout the department.

ED physicians performed test ordering further along the patient journey through the department and sometimes based their ordering decisions on results already returned from tests ordered at an earlier stage. Additional tests could be added to an existing blood sample, or additional blood draws might be required. Venesection was commonly performed by physicians and nurses and, in less common cases, by a limited number of technical assistants employed by the department. A process model of the laboratory test ordering process in the ED is presented in Figure 2. Participant-reported issues with current test ordering workflows, decision-making requirements, and expectations for an electronic decision-support tool are published elsewhere [19]. In summary, a data-driven dashboard approach to supporting decision-making was raised as a potentially helpful tool, in lieu of guidelines, as peer behavior was reported to have some influence on decision-making. Respondents also requested that any intervention should not prolong or introduce additional steps to the workflow, should be fully integrated into existing systems, and should not impose restrictions on ordering behavior.

**Figure 2.**
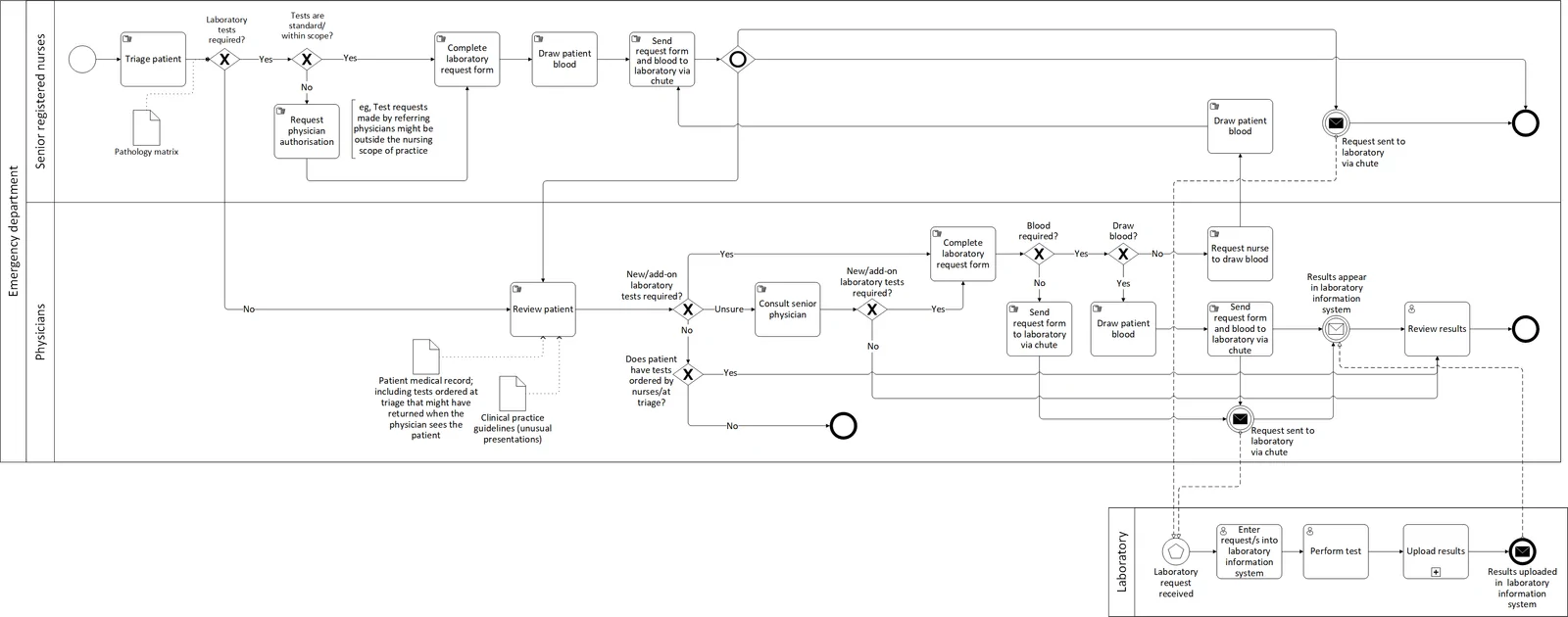
Emergency department (ED) laboratory test ordering process.

### The ED-POST (Phase 1: Development)

The results from the phase 1 exploration were presented to the digital developers and synthesized into a prototype development document (Multimedia Appendix 2), which was shared with the same developers. Table 3 presents an excerpt of the document and shows how findings from phase 1 were operationalized in the design of ED-POST. The ED-POST prototype was then developed by a data scientist, and the user interface was designed by a member of the university research team using Tableau Software LLC (Professional Edition version 2023.2.1). The ED-POST tool drew upon the Australian Pathology Atlas of Variation (Atlas) data source, containing over 8 million encounters and 25 million tests across 4 years in 35 facilities [28,29]. The Atlas analytical model merges ED data such as presenting problem, patient demographics, and triage categories with test ordering profiles. It also measures ED throughput and pathology turnaround times to identify intervals around patient arrival in the ED to triage, arrival to pathology order, the overall length of stay, and mode of separation. This information can be used to create probability scores from 0% to 100% for the likelihood of tests being ordered in scenarios with known variables at the time of triage (presenting problem, triage category, age, sex, and facility).

**Table 3.** Operationalization of phase 1 findings.

Issue/objective	Example quotes	ED-POST^a^ feature
Reduce overordering of tests	*“*We probably just don’t get the proportion of common tests right. So more than half of our patients should receive some kind of blood testing and probably it shouldn’t be 60%, it probably should be 40% or something like that.…we’re ordering full blood count, UACs^b^ on everyone and we don’t need to.”* * *“*[Nurses] often take a lot of liberties and we’ll add on things like a coagulation profile or an INR^c^ on someone because they’re on Apixaban. And it’s completely redundant information. It means nothing. It doesn’t tell us anything helpful.”	Display of departmental ordering trends and those of other EDs^d^, as well as the state average to allow for the comparison with peer hospitals and the overall benchmark
Address inadequate ordering	“I think it’s important to show whether you’re an outlier,...another example of that would be...where we are ordering inadequate numbers of fibrinogen in major trauma patients. Whereas actually we should be ordering them in all major trauma patients with hemorrhagic shock.”	Display of departmental ordering trends and those of other EDs, as well as the state average to allow for the comparison with peer hospitals and the overall benchmark
Influence clinician ordering behavior	“senior clinicians are able to attempt to influence the behavior of the broad medical group and nurses essentially, if they’re aware of information about this....And one of the things that would allow us perhaps to make changes is an active dashboard of current ordering behavior....just the general ordering pattern would be useful for the more senior doctors and the ones who are clinical leaders and that they can then influence.” “clinical culture is mostly influenced by doctors watching what each other do. So if you guys are going to tell us what all other doctors are doing, we’re probably just going to do that.” “If it happened that I ordered a test and it says nobody else orders this test, then it would definitely make me reflect on my practice.”	Display of departmental ordering trends to allow for the comparison with peers and facilitate quality improvement
Minimize workflow disruption	“It needs to be a system where you can access it, you can log on and you can do it quickly.” “if you can find a way to present the information to people in a way where it doesn’t slow them down or get in their way, you probably would find that...you’d see some differences in test ordering behavior.” “the most benefit to it would be if it’s embedded within an electronic ordering system because then you’re doing it at the same time. Like it’s helping you guide your decision making as you go”	Full integration with EMR^e^ The ED-POST prototype will appear as a smaller passive reference screen on the EMR ordering page and will not impose ordering restrictions or require acknowledgment (eg, via extra clicks)

a ED-POST: Emergency Department Electronic Laboratory Test Ordering Decision Support Tool.

b UAC: urine albumin-to-creatinine ratio.

c INR: international normalized ratio.

d ED: emergency department.

e EMR: electronic medical record.

ED-POST uses patient triage variables to calculate the probability that the top 11 most commonly ordered laboratory tests in the ED will be ordered based on patients with an identical presenting profile from its dataset of 8 million encounters. The tool then displays ED ordering trends for a specified hospital (selectable via a drop-down menu) in comparison with the state average as a means of supporting test ordering decision-making (Figure 3).

**Figure 3.**
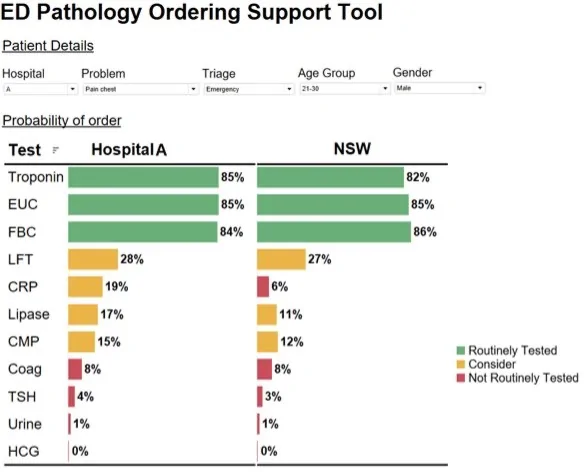
Screenshot of Emergency Department Pathology Ordering Support Tool (ED-POST). CMP: comprehensive metabolic panel; CRP: C-reactive protein; ED: emergency department; EUC: electrolytes, urea, and creatinine; FBC: full blood count; hCG: human chorionic gonadotropin; LFT: liver function tests; NSW: New South Wales; TSH: thyroid-stimulating hormone.

### Outcomes of ED-POST Demonstration (Phases 2 and 3: Interviews and Forum)

#### Impact of Ordering Role on the Perceived Use and Value of the ED-POST Tool

Interview respondents’ perceived use and value of the ED-POST tool following the prototype demonstration were found to vary depending on their role in the laboratory ordering process. Triage nurses and junior physicians who were more involved in ordering common tests early in the patient encounter saw greater utility in the tool as a resource or guide in their routine clinical practice.

*I think it would be helpful. Maybe make things a bit quicker.…By recommending what we should order and using it as a guideline to direct staff on what should be ordered.*

[NB1, triage nurse]

*…it would be good to be reminded…if there were tests that at a certain triage…Some investigations can escape my mind…and if I find that there’s a commonly ordered test for a specific presentation and I could be reminded to order that test early.*

[DG1, intern]

Furthermore, the ED-POST tool was seen by interviewees as useful for uncommon presentations and was more likely to influence ordering behavior when individual decisions considerably differed from the outputs displayed by the tool.

*I’d have a second think of it. It’s useful especially for something that’s less typical.*

[DE1, intern]

*if there was a test that where our averages or my personal average would be much, much higher than the rest of the state, I’d be less likely to add that on.*

[DG1, intern]

*It would make me think why I’m ordering a CMP [Comprehensive Metabolic Panel]…it would make me think that do I actually need a CMP and what’s it going to tell me and am I requesting it out of just force of habit potentially. So it could change practice a little bit in that respect.*

[DD1, registrar]

Senior physicians who were more likely to order add-on tests and review patients at a later stage of the ED encounter saw less utility of the tool in their daily practice for a number of reasons. They explained that common or standard tests were often already ordered (sometimes with results already returned) by the time they encountered the patient. Unusual diagnoses often presented with common symptoms and, once determined to be rare presentations, would require ordering uncommon tests that were not included in the ED-POST tool.

*[senior physicians] get very little opportunity in this ED to initially fill out the blood test because they’re largely performed at triage by the CIN [Clinical Initiative Nurse] nurses….So I think from a doctor’s point of view the tests are often performed before we get to see the patients so they’re ready for us.*

[DH1, staff specialist]

[Regarding unusual presentations]: *the nurses at the front will come to us and say, “got this patient with these weird signs and symptoms, what would you recommend?” And then that’s never going to be covered in this kind of [system].*

[DI1, staff specialist]

#### Occupational Hierarchy, Role-Based Priorities, and Their Broader Implications for the ED-POST Tool

In contrast to the roles of junior physicians and nurses who more frequently ordered tests as part of direct patient care, senior physicians’ views of the ED-POST tool reflected the broader aspects of their role in departmental coordination, oversight, and management that influenced overall ED functioning. Most notably, the big data-driven quality of the tool was perceived to be useful for education and departmental quality improvement.

*I could probably perhaps use it as a training tool for the CIN nurses to say look, when a patient has this, can you do these green blood tests because they’re done by everybody and they’re the same as the state average but avoid these orange and red tests […] I think it would be useful to show this to the nurse educators to think if they would find it useful in training those front of house triage and CIN nurses.*

[DH1, staff specialist]

*I think what is interesting to see is some of the tests that we do order compared to the average, it’s good to highlight that because there are certain things like the urine test that we do on everybody in this ED that is massively over performing and I think that could highlight that we shouldn’t be doing that. That’s been a big problem to stop people testing people’s urine routinely here.*

[DH1, staff specialist]

Senior ED physicians in the stakeholder forum largely echoed the sentiments of the senior physician interviewees, expressing limited perceived value of the tool in assisting their test ordering practices, but supported its use by nurses who perform the majority of test ordering at the beginning of the patient encounter in the ED. Similarly, they recognized the capacity of ED-POST to provide feedback on departmental ordering patterns and its ability to highlight issues and initiate remediation efforts. To this end, they advocated for a shift toward a real-time dashboard of departmental ordering trends or a similar ability to generate regular reports that could facilitate discussions at quality improvement meetings. Both would enable the identification of outlying ordering behaviors or waste and provide evidence of the efficacy of remedial interventions. In addition to providing a departmental overview, the ability to report on individual ordering trends was also suggested to better tailor education, training, or interventions to specific teams or staff groups.

#### Drivers of Test Ordering Behavior

Senior clinicians across staff categories at the study site and at the stakeholder forum questioned whether common behavior reflected the gold standard of practice, preferring instead a digital tool that offered advice based on evidence or state-endorsed guidelines as a more authoritative and effective influence on test ordering decision-making among junior colleagues. In the same vein, the integration of information regarding the utility of each test, displayed for each patient profile, was also suggested.

*We should be going off evidence-based practice…From a Nurse Practitioner point of view, unless it’s increasing my knowledge or giving me reasons, it’s not really doing anything*

[ND1, transitional nurse practitioner]

Senior physicians in the stakeholder forum were concerned that the display of departmental trends in ED-POST was more likely to encourage overordering among junior physicians by acting as a visual prompt, even when probability scores did not depict a great majority. For instance, a score of 65% likelihood of a test being ordered might be sufficient to encourage a junior physician to order a test when it previously would not have been ordered. Additionally, the reliability of a statistically derived state average was debated among forum members, with senior physicians concerned that the calculation would include all outlying hospitals when only one outlying hospital might represent the true gold standard, thereby misrepresenting best practice and negatively influencing behavior.

#### The Real Cost of Laboratory Testing

Furthermore, senior physician interviewees and forum participants queried the possibility of displaying cost information per test (with a distinction between initial order and add-on costs) as a means of prompting consideration and rational ordering. However, the laboratory-based data scientist participant in the forum explained how laboratory tests were billed, emphasizing that the monetary cost of additional tests might not be as high as is commonly believed (eg, add-on tests did not incur any additional charge). Rather, they stressed the need to balance the cost of a small number of additional tests with the broader public health costs of extra patient hours in the ED if relevant tests were not ordered in a timely manner.

## Discussion

### Principal Findings

A multimethod qualitative co-design approach to understanding clinician workflows illuminated the different roles of various clinical groups in laboratory test ordering in the ED and how their decision support requirements were influenced by their role. Triage nurses who ordered preliminary and routine tests at the beginning of the patient encounter were more likely to use ED-POST as a point-of-ordering decision support tool. Junior physicians also saw value in ED-POST as a form of backup to reinforce individual impressions, as a reminder, and as a guide in cases of uncommon presentations. Senior physicians responsible for determining a differential diagnosis at a later stage of the patient journey through the ED saw less utility in ED-POST for supporting their ordering decisions but recognized potential in its big-data synthesis capabilities to support the departmental management component of their role and quality-improvement activities.

### Implications

The “one-size-fits-all” fallacy viewpoint of Karsh et al [30] identified the need to acknowledge the substantially different clinical roles extant in a health setting and the differing digital health needs of individual practitioners. Findings of this study indicate that the design of department-wide interventions should account for individual, as well as the collective, requirements of the broader team. After studying workflows in the ED setting during transition from paper medical records to an EMR, Park et al [31] identified the need for digital systems to support both the collective needs and roles and responsibilities of individual medical staff. Our study highlighted the effect of individual roles in test ordering and hierarchical role-based priorities on the perceived use and the impact of a digital health tool within a department.

The validity of departmental trends and statistically derived state averages as benchmarks for guiding test ordering behavior was a key criticism that was raised in this study, with many participants expressing a preference for evidence-based advice. The current digital health landscape is saturated with laboratory test ordering guidelines [15,16], guideline-based alerts and decision support [32], and EMR-based care sets and pathways [33], which have been implemented with varying degrees of success. One study of note by Leis et al [34] on admission order sets reported an increased likelihood of overordering, despite being designed to improve guideline adherence. The authors found that increased visibility of tests included in an order set can act as a behavioral “nudge,” influencing clinician decisions independently of clinical needs. This is relevant to concerns raised by clinical forum participants in this study, who feared that the increased visibility of common tests via ED-POST might encourage overordering by junior physicians, even when probability scores did not depict a substantial majority.

While existing interventions typically provide general and generalizable advice, the distinctiveness of ED-POST is its ability to harness site-specific data to highlight local issues and facilitate quality improvement via the mechanism of tailored feedback. A small number of studies examining feedback-based interventions to address unnecessary or low-value laboratory test ordering have reported positive outcomes [35]. However, such interventions were temporary and relied on manual processes, and there are no current studies reporting feedback-based electronic decision support at the point of ordering. Furthermore, all studies concluded that test ordering stewardship interventions were most effective when feedback was implemented in conjunction with other approaches, most commonly clinician education [35,36]. Given the positive reception to the big data-driven properties and quality improvement potential of ED-POST, the efficacy of the tool might be better achieved if implemented as part of a comprehensive suite of test ordering stewardship measures. The continued development of ED-POST’s real-time or near–real-time data synchronization potential and granular reporting of ordering trends at the staff group or individual level could inform targeted departmental quality improvement activities in addition to the implementation of a point-of-ordering decision support tool alone.

Finally, discussions during the stakeholder forum suggested a lack of clarity regarding the costs and charges incurred and imposed by the laboratory, respectively. A prominent argument against the overordering of tests from senior ED physicians in this study revolved around the issue of cost. However, the true cost of laboratory tests encompasses more than the direct dollar value attributed to the test, with add-ons, in particular, requiring additional resources and potentially creating delays in the testing process [37]. Increased transparency regarding laboratory billing structures and evidence between the *real* cost of tests against the potential cost of a delayed patient disposition in cases of underordering might reshape overarching attitudes towards the ordering of additional and add-on tests [38].

### Limitations

This study reports user perceptions of an electronic laboratory ordering support tool without quantitative or observational measures of usability, utility, or effectiveness, and outcomes in actual practice might vary. The study was also limited because it did not include the entire population of clinicians involved in laboratory test ordering in the department and was largely a single-site study, limiting transferability to other settings. However, the multimethod, qualitative co-design approach resulted in the in-depth exploration of clinical roles and pathology test ordering workflows within the departmental context and input from representatives from 3 hospital sites.

### Conclusions

Requirements for laboratory test ordering decision support can vary substantially across clinical roles within a department. Understanding roles and workflows in the early developmental stages of a digital health tool, and involving users in every phase of development, are necessary to optimize adoption, scalability, and outcomes. Future design and implementation efforts should prioritize role-sensitive, workflow-integrated systems. Incorporating timely, feedback-driven mechanisms using local, context-specific data may facilitate more informed test ordering behaviors. Importantly, digital interventions should support both individual clinical decision-making and broader departmental objectives.

## Supplementary material

10.2196/92744 Multimedia Appendix 1 Interview and discussion guides.

10.2196/92744 Multimedia Appendix 2 Prototype development document.

## Abbreviations

BPMN: Business Process Model and Notation
CIN: clinical initiatives nurse
CMP: comprehensive metabolic panel
ED: emergency department
ED-POST: Emergency Department Electronic Laboratory Test Ordering Decision Support Tool
EMR: electronic medical record
