# Supplementary material for: Reducing Unwarranted Variation in Emergency Department Laboratory Test Ordering With a Feedback-Based Decision Support Tool: Qualitative Formative Evaluation
Source: JMIR Form Res. 2026 Nov 13;10:e92744. doi: 10.2196/92744 (PMC13765780; doi:10.2196/92744)
Supplement: Supplementary file 1 [file formative-v10-e92744-s001.docx]

**Phase 1**

Interview Questions

1. What is your role in the ED?
2. What is the current laboratory ordering process in this ED?
3. What resources (if any) do you rely on in your test ordering decision making?
4. What are your expectations for a digital laboratory test ordering decision support tool?

**Phase 2**

Interview Questions

1. How useful do you think this tool would be for your ordering work practices?
2. Do you think it will affect your workflow?
   a. Speed/efficiency
   b. Sequence/coordination/number of steps
3. Do you think the information presented in the tool will affect your decision-making?
4. What would you like to see improved/changed?

**Phase 3**

Discussion topics

1. [Following demonstration of ED-POST] What are your thoughts of the ED-POST tool?
2. [Following presentation of Phase 2 feedback] What are your thoughts on feedback from [the study site]? Do they resonate (or not) with your experiences of your department?
