# Supplementary material for: Reducing Unwarranted Variation in Emergency Department Laboratory Test Ordering With a Feedback-Based Decision Support Tool: Qualitative Formative Evaluation
Source: JMIR Form Res. 2026 Nov 13;10:e92744. doi: 10.2196/92744 (PMC13765780; doi:10.2196/92744)
Supplement: Supplementary file 2 [file formative-v10-e92744-s002.docx]

| **Stakeholders** | | | |
| --- | --- | --- | --- |
| **Stakeholder/Position** | | | **Role** |
| Senior Emergency Physicians/Consultants | | | Subject matter experts  End user  Clinical thought leaders and champions |
| Emergency intern/resident/registrar/JMO | | | End user |
| Registered nurses | | | End user |
| Local Health District Pathology Director | | | Subject matter expert |
| Data Scientist - statewide public pathology service | | | CDS-specific/general IT |
| Associate Director Planning and Performance - statewide public pathology service | | | CDS-specific/general IT |
| **Goals** | | | |
| **Quality Use of Pathology** | | | |
| **System/Program Objectives** | **Clinical Objectives** | **Quotes** | |
| Prevent errors of commission | - Reduce inappropriate ordering of tests - Foster compliance with best practice guidelines/evidence-based practice - Improve appropriateness of overall workup and treatment plans | *“the majority of the time […] it's done on full autopilot and you don't even look at the form that you are circling.  You just, I want a full blood count.  You push that button and you walk away.”*    *“a good idea is to have more rational test ordering”*    *“We probably just don't get the proportion of common tests right.  So more than half of our patients should receive some kind of blood testing and probably it shouldn't be 60%, it probably should be 40% or something like that. […] we're ordering four blood count UACs on everyone and we don't need to.”*    *“batch ordering just increases testing, which is often probably not beneficial for the patient”*    *“[Nurses] often take a lot of liberties and we'll add on things like a coagulation profile or an INR on someone because they're on Apixaban.  And it's completely redundant information.  It means nothing.  It doesn't tell us anything helpful. The only times we need a coag profile is if you want an INR on someone on Warfarin. But there's nothing that matters clinically about coag levels on someone on apixaban”*    *“we have a clinical guideline document that underpins what the nurses should be doing.  They're just not following the guideline all the time.”*    *“the most obvious solution, I suppose, to have some capacity built into the IT infrastructure when you log in that says, you're [a provider], […] you have this ordering profile within the system, which means you can order these kinds of bloods. […]I think if we were transitioning onto electronic ordering platform, the only way to ensure that they're working within the pre-approved scope that the nursing staff have would be to put those restrictions on what they can order.”* | |
| Prevent errors of omission | - Attention to clinically significant laboratory results - Improve appropriateness of overall workup and treatment plans - Address inadequate ordering | *“They often take a lot of liberties and we'll add on things like a coagulation profile or an INR on someone because they're on apixaban.  And it's completely redundant information.  It means nothing.  It doesn't tell us anything helpful.”*    *“I think it's important to show whether you're an outlier, […] another example of that would be […]  where we are ordering inadequate numbers of fibrinogen in major trauma patients.  Whereas actually we should be ordering them in all major trauma patients with haemorrhagic shock.  And so if it's said I was an outlier there, then that's because we are inadequately ordering that test.”* | |
| **Decision Making** | | | |
| Address clinicians’  ordering information needs | - Feedback on departmental ordering patterns in the format of a dashboard to inform and facilitate evaluation of department-specific behaviour-change campaigns - Provide feedback on individual ordering practices as compared with colleagues | *“senior clinicians are able to attempt to influence the behaviour of the broad medical group and nurses essentially, if they're aware of information about this.  So the more information we get, the better.  And one of the things that would allow us perhaps to make changes is an active dashboard of current ordering behaviour.  So taking an example of coagulation studies, if we attempt to decrease their use because we perceive them to be wasting money by undertaking some kind of information campaign or posturing or something like that, if we can see that that's working in real time, because we get information back every week and we see coagulation studies drop and we see that there's cost saving, then that will reinforce that behaviour. […] I think just the general ordering pattern would be useful for the more senior doctors and the ones who are clinical leaders and that they can then influence.  If they can see that they're making changes as produced improvements, they'll continue to try and drive that.”*    *“clinical culture is mostly influenced by doctors watching what each other do.  So if you guys are going to tell us what all other doctors are doing, we're probably just going to do that.”*    *“That may reduce some over-ordering if doctors are ordering exotic tests and they're made aware of the fact that they're unique.  That will tend to reduce that.”*    *“I think it's important to show whether you're an outlier, but I also think that there'll be tests that won't come up as an outlier.  For instance, a troponin is ordered on people that don't have chest pain and that's really irrelevant. But I think a large portion of people will be ordering troponin in the wrong setting.  And if you order it, it probably looks like you're fine because lots of other people have also ordered it.  So I think it's useful if I've ordered a test and nobody else would order the test in those situations, I'd be reflective of my practice.  […] another example of that would be the other way around where we are ordering inadequate numbers of fibrinogen in major trauma patients.  Whereas actually we should be ordering them in all major trauma patients with haemorrhagic shock.  And so if it's said I was an outlier there, then that's because we are inadequately ordering that test.”*    *“Might reassure me if I'm practicing a way that's wasteful and everybody else is practicing in a way that is also wasteful.”*    *“If it happened that I ordered a test and it says nobody else orders this test, then it would definitely make me reflect on my practice.”*    *“I think it's really helpful for junior doctors that haven't been doing it a long time and aren't familiar with ordering blood tests.  I think it would be really helpful if you were starting as an intern or resident in the emergency department to receive some feedback with a tool that could guide you to say like, this seems like you're doing good thing.  Like, this seems like a reasonable test to order for this presentation and only ordered for this presentation.  Or alternatively guide you in the other direction to say, this is not commonly ordered for this presentation.  And that could flag whether they need to ask for help.”*    *“I think it would be very helpful for junior clinicians, but I wonder about senior clinicians, how much it would help them or how much it would change their practice.”* | |
| Optimise choice of laboratory tests based on additional considerations such as cost of test | - Reduce unnecessary laboratory tests - Optimise cost-effectiveness of care | *“it might be quite useful for them to look up a test and see that it seldom makes any difference or that it costs a lot of money.”*    *“Whether it really changes behaviour is hard to tell, but the more you make anyone aware of the cost of something, as economists know, you will produce some behaviour change.”*    *“there's an increase in trend in clearly the nurses taking those liberties I mentioned with the matrix that they're supposed to work within of trying to be helpful, but ordering things that actually don't really have any clinical relevance at all.  And they cost us money.  That's the angle everyone always has, why are we adding these tests on, or why are we doing these tests when it's not telling us anything and it's costing the department money per test.”* | |
| **Care Processes** | | | |
| Improve delineation of responsibility for test results (e-ordering in general) | - Clearer tracking/documentation of ordering clinician - Support handover processes - Improve safety of test result follow-up | *“with the paper, it's essentially impossible to figure out who it is [who has ordered][…]There's fairly little chain of responsibility possible with the paper system at the moment”* | |
| Optimise efficiency of workflow | - Integration with existing context/workflow/systems/   infrastructure | *“The biggest pressure on ordering is to get it done quickly so it doesn't cause a delay”*    *“It needs to be a system where you can access it, you can log on and you can do it quickly. […] it's been worldwide found that e-ordering and e-records slow everything down”*    *“if you can find a way to present the information to people in a way where it doesn't slow them down or get in their way, you probably would find that there's, at least on a big scale anyway, you'd see some differences in test ordering behaviour.”*    *“there can be no free computers in the department at times”*    *“It's hard enough to get access to a computer […] for the nursing staff out the front”*    *“the most benefit to it would be if it's embedded within an electronic ordering system because then you're doing it at the same time.  Like it's helping you guide your decision making as you go”*    *“As long as it doesn't make it harder. […] we've been through this with our online medical prescribing and we've removed it from the emergency department because it's been dangerous.  We have a resuscitation patient that needs lots of medication given in a timely manner to find a computer space to be able to log on and then click many, many buttons to legally prescribe something so the nurses can legally give it in that order will delay their resuscitation of a patient.  So we've gone back to paper charts in that setting so that we can do that legally and timely.”*    *“I just hope that when a system comes through that's really carefully discussed with users, because the time it takes for us to write each form is important because if we are writing forms as a consultant on 20 or 30 people, and if you've changed the system from taking 15 seconds to a minute, then that's a lot of time.  We are doing a lot of things at once.  So the ease of us making that happen, the time it's going to take us is important because it will add to our workflow.”*    *“the more clicks you make us do, the more time we can't go to patient care, and we are already accommodating that and time pressed.”*    e-ordering in general:    *“in our emergency department, I think there's been a quite a big push to keep it on paper, our pathology ordering, because it's seen as being quicker.  In a pathology request form, you can make your assessment.  You can do the paper really quickly, and then that's that encounter complete, whereas different emergency departments have the electronic prescribing on all things.  It just takes a little bit more time.  It's just such a time poor environment that I wonder the utility, but in this emergency department.”*    *“we had electronic medication prescriber here a few years ago that was overturned after a short period of time because they felt that it added a lot of time to encounters and also took longer for patients to get time sensitive medications because it extended things more”*    *“technically I think it would make it safer for patients.  I think it would reduce errors and potentially reduce unnecessary ordering, especially if you coupled alongside a system that can support you to say, this is often ordered for this presentation, et cetera.  But I think it also could come at the negative of just being, it's such a time poor environment.”* | |
